# Levels of Plasma N-terminal Pro-brain Natriuretic Peptide and D-dimer on the Prognosis of Patients with Acute Cerebral Infarction

**DOI:** 10.12669/pjms.344.14513

**Published:** 2018

**Authors:** Rui Wang, Yamin Wei, Junfang Teng

**Affiliations:** 1 Rui Wang Neurological Intensive Care Unit, The First Affiliated Hospital of Zhengzhou University, Zhengzhou, Henan, 450000, China; 2 Yamin Wei Neurological Intensive Care Unit, The First Affiliated Hospital of Zhengzhou University, Zhengzhou, Henan, 450000, China; 3 Junfang Teng Neurological Intensive Care Unit, The First Affiliated Hospital of Zhengzhou University, Zhengzhou, Henan, 450000, China

**Keywords:** Cerebral infarction, NT-pro BNP, D-dime

## Abstract

**Objective:**

To investigate the effects of levels of D-dimer and N-terminal pro-brain natriuretic peptide (NT-pro BNP) on the prognosis of patients with acute cerebral infarction.

**Methods:**

One hundred and twenty-four patients with acute cerebral infarction who were admitted to the hospital between July 2014 and July 2016 were selected as the observation group; 100 normal people who had health examination in the center of physical examination of our hospital were selected as the control group. The levels of D-dimer and NT-pro BNP of the two groups were observed; the correlation between the levels of plasma NT-pro BNP and D-dimer and area of cerebral infarction, complications and death condition of the observation group was investigated.

**Results:**

The levels of D-dimer and NT-pro BNP of the observation group were much higher than those of the control group, and the difference was statistically significant (P<0.05). The levels of D-dimer and NT-pro BNP of the observation group were significantly higher than those of the control group, and the difference had statistical significance (P<0.05). The levels of plasma NT-pro BNP and D-dimer of the patients with disturbance of consciousness and high blood pressure were apparently higher than those with no disturbance of consciousness and normal blood pressure, and there was a statistically significant difference (P<0.05). The patients were followed up for half a year. The levels of D-dimer and NT-pro BNP of the dead patients were much higher than those of the survived patients on admission.

**Conclusion:**

The levels of plasma NT-pro BNP and D-dimer can reflect the disease condition and prognosis of patients with acute cerebral infarction. Higher levels of NT-pro BNP and D-dimer indicates poorer prognosis. This work can provide a guidance for the clinical treatment of acute cerebral infarction.

## INTRODUCTION

Cerebral infarction, a common cerebrovascular disease in clinic, is induced by local ischemia and necrosis of cerebral tissues. It is featured by sudden attack, high incidence of complications, high disability rate, high death rate and poor prognosis.^1^,^2^ With the changes of living habits in recent years, the incidence of cerebral infarction shows a tendency of obvious increase.^3^ Some patients with cerebral infarction have poor recovery after treatment. Therefore, more effective diagnostic indicators are needed to provide an important significance for clinical treatment.

In recent years, Brain Natriuretic Peptide (BNP) and D-dimer have important significance in the occurrence of cerebral infarction and were closely correlated to the occurrence of cerebral infarction. BNP is not stable in plasma because of its short half-life period.^4^,^5^ N-terminal pro-BNP (NT-pro BNP) is characterized by long half-life period and easy detection. NT-pro BNP is more stable than BNP in plasma. Hence it can be replaced by NT-pro BNP.^6^ This study investigated the effects of levels of D-dimer and NT-pro BNP on the disease condition and prognosis by detecting the levels of D-dimer and NT-pro BNP of the patients with acute cerebral infarction.

## METHODS

One hundred and twenty-four patients with acute cerebral infarction who were admitted to the hospital between July 2014 and July 2016 were selected and set as an observation group. One hundred healthy people were selected as a control group. The study protocol was approved by the local ethics committee. In the observation group, there were 69 males and 55 females; they aged from 52 years to 78 years (average (67.4±7.2) years). In the control group, there were 60 males and 40 females; they aged from 51 to 77 years (average (68.2±8.1) years). The difference of gender and age between the two groups had no statistical significance (P>0.05). Therefore the results were comparable.

### Inclusive criteria

Patients who had acute cerebral infarction that was diagnosed according to the Essentials of Diagnosis of Cerebrovascular Diseases which were approved by the Fourth Academic Conference of Cerebrovascular Diseases (1995) and based on the results of head Computed Tomography (CT) or Magnetic Resonance Inspection (MRI) and were admitted to the hospital within 24 hour after attack were included.^7^ The research subjects in the two groups were informed about the objective of the study and signed informed consent was obtained when they were awake.

### Exclusive criteria

Patients who had heart, renal and respiratory function failure, tumor or severe infection, underwent surgical operation in recent three months before grouping, had myocardial infarction and (or) other vascular occlusion diseases previously, had autoimmune diseases previously, or took inflammation inhibition drugs or immunosuppressors were excluded.

### Research methods

The data of all the subjects including gender, age, history of present illness and past medical history were collected. 8ml of elbow venous blood was collected on the second day after admission and centrifuged at 3000 r/minutes for 15 minutes to separate plasma. The level of NT-pro BNP was detected using a fully automatic electrochemistry luminescence immunity analyzer (Cobase4112010; Roche Inc., China); the level of D-dimer was detected using immumofluorescence method.

### Observation indexes

The levels of NT-pro BNP and D-dimer were compared between the two groups. The correlation of the area of cerebral infarction and complications with levels of NT-pro BNP and D-dimer of the patients in the observation group was observed. The condition of prognosis was observed by following up the patients in the observation group for half a year. The levels of the above indexes of the patients with different prognosis on admission were analyzed. According to the classification criteria of cerebral infarction,^3^ if the diameter of a lesion of cerebral infarction was larger than four cm or a lobe, then it was considered as massive cerebral infarction; if the diameter of a lesion was no larger than four cm, then it was considered as small-area cerebral infarction.

### Statistical processing

Data were statistically processed using SPSS ver. 20.0. Measurement data were expressed as mean±standard deviation. Comparison between groups was performed using t test. Comparison between enumeration data was performed using Chi-square test. Difference was considered as statistically significant if P<0.05.

## RESULTS

### Comparison between plasma NT-pro BNP and D-dimer between the observation group and the control group

The levels of NT-pro BNP and D-dimer of the observation group were significantly higher than those of the control group, and the differences had statistical significance (P<0.05; Table-I).

**Table-I:** Comparison of plasma NT-pro BNP and D-dimer between the two groups

Group	N	NT-pro BNP (ng/L)	D-dimer (mg/mL)
Observation group	124	2358.17±921.73	364.52±34.84
Control group	100	133.5±46.8	137.6±40.5
t		10.716	2.517
P		<0.05	<0.05

### Analysis on correlation between different areas of cerebral infarction and different indexes

The levels of plasma NT-pro BNP and D-dimer of the patients with massive cerebral infarction were significantly higher than those of non-massive cerebral infarction, and the differences had statistical significance (P<0.05; Table-II).

**Table-II:** Comparison of levels of plasma NT-pro BNP and D-dimer between patients with different areas of cerebral infarction

Group	N	NT-pro BNP (ng/L)	D-dimer (mg/mL)
Massive cerebral infarction group	67	3827.26±1027.31	517.48±567.34
Non-massive cerebral infarction	57	229.03±218.55	163.75±143.77
t		16.518	3.905
P		<0.05	<0.05

### Analysis on the correlation between complications and different indexes

Forty-eight patients had disturbance of consciousness, and 76 patients had no disturbance of consciousness. Eighty-seven patients had hypertension, and 37 patients had no hypertension. The levels of plasma NT-pro BNP and D-dimer of the patients with disturbance of consciousness were (5034.58±5137.26) ng/L and (164.13±123.74) mg/mL respectively, which were higher than (456.83±533.26) ng/L and (164.13±123.74) mg/mL of the patients without disturbance of consciousness respectively; the differences had statistical significance (P<0.05). The levels of NT-pro BNP and D-dimer of the patients with hypertension were (4184.35±7563.31) ng/L and (544.43±572.08) mg/mL respectively, which were higher than (919.63±327.66) ng/L and (147.93±115.31) mg/mL of the patients without hypertension; the difference were statistically significant (P<0.05).

### Effects of levels of plasma NT-pro BNP and D-dimer on prognosis of patients with cerebral infarction

levels of plasma NT-pro BNP and D-dimer of the death group were apparently higher than those of the survival group, and the differences were statistically significant (P<0.05; Table-III).

**Table-III:** Comparison of QLQ-C30 scale score between two groups

Group	N	NT-pro BNP (ng/L)	D-dimer (mg/mL)
Death group	22	9134.37±10246.29	920.06±957.07
Survival group	102	669.13±743.18	223.54±207.16
t		5.426	4.386
P		<0.05	<0.05

## DISCUSSION

Acute cerebral infarction which is frequently seen in clinics is featured by sudden attack and rapid development and has high disability rate and fatality rate.^8^ With the constant enhancement of living level, the incidence of cerebral infarction has significantly increased, which has become a focus. Though the determination of severity of cerebral infarction depending on the imaging examination such as CT and MRI has certain values, the evaluation on clinic treatment is limited and the evaluation on prognosis is insufficient. Hence searching effective prognosis evaluation means has been one of the important step in the clinical treatment of acute cerebral infarction.^9^,^10^

With the constant development of medicine, NT-pro BNP and D-dimer is found being closely correlated to the occurrence and development of cerebral infarction. NT-pro BNP, a fractured fragment of BNP, is featured by long half-life period and easy detection compared to BNP.^11^ Studies have found that NT-pro BNP has important significance in cerebrovascular diseases.^12^,^13^ In this study, the level of NT-pro BNP of the observation group was significantly higher than that of the control group, which was consistent with the results of the current studies. BNP can promote natriuresis, inhibit secretion of antidiuretic hormone and sympathetic nerve impulse, and regulate baroreceptor. Most of the studies have suggested that the level of NT-pro BNP of patients with acute cerebral infarction was higher than that of normal people. Though the mechanism of increase of the level of NT-pro BNP has not been thoroughly clarified, some scholars thought it might be contributed to local ischemia and hypoxia of cerebral tissues, abnormal secretion of nerve transmitters induced by strong stimulation on hypothalamo-hypophyseal system or tension of blood vessel wall induced by hemodynamic changes.^14^,^15^ A previous study has demonstrated that the level of plasma NT-pro BNP of patients with hypertension was higher than that of patients without hypertension,^16^ indicating that blood pressure could affect hypothalamo-hypophyseal system and induce the abnormal secretion of nerve transmitters to increase the secretion of plasma NT-pro BNP. It was also proved in this study.

The blood coagulation and activation of fibrinolytic system of patients with acute cerebral infarction can be reflected by the changes of the level of D-dimer. D-dimer is the main marker for reflecting the degree of thrombolysis.^17^ When blood coagulation occurs, a large number of D-dimer compounds generate after fibrin degradation, which induces the concentration increase of D-dimer.^18^ D-dimer has high stability and sensitivity; hence its concentration in blood will not be affected by other factors and thereby can accurately evaluate cerebral infarction condition of patients. The results of this study has demonstrated that the level of D-dimer of the patients in the observation group was significantly higher than that in the control group, indicating the role of D-dimer in the occurrence and development of cerebral infarction. Increase of levels of D-dimer and NT-pro BNP is because of vascular injury, platelet activation, clotting mechanism hyperfunction and changes of blood flow state and will not be affected by jaundice and hemolysis. The larger the area of cerebral infarction is, the more active coagulation function and fibrinolytic system are and the higher the levels of D-dimer and NT-pro BNP is.^19^,^20^ This study found that the levels of D-dimer and NT-pro BNP of the patients with massive cerebral infarction were obviously higher than those of the patients in the control group, indicating that patients with severer cerebral infarction had higher levels of D-dimer and NT-pro BNP. It was also found that the levels of D-dimer and NT-pro BNP of the death patients were much higher than those who survived after half-year follow up. Moreover, the patients with higher levels of D-dimer and NT-pro BNP had poorer prognosis.

## CONCLUSION

Levels of plasma NT-pro BNP and D-dimer have important clinical significance in determination of disease condition, classification of severity, treatment guidance and prognosis prediction. After some heart and lung diseases, renal failure and liver cirrhosis can also induce the increase of levels of plasma NT-pro BNP and D-dimer are excluded. It can be an efficient, convenient and effective test indicator for the diagnosis and treatment of cerebral infarction. The clinical values of levels of plasma NT-pro BNP and D-dimer for cerebrovascular diseases should be given more attention in future.

### Authors’ Contribution

**RW:** Study design, data collection and analysis.

**RW & YMW:** Manuscript preparation, drafting and revising.

**JFT:** Review and final approval of manuscript.

## References

[1] Zhang X, Wu J, Zhang B (2015). Xuesaitong injection as one adjuvant treatment of acute cerebral infarction:a systematic review and meta-analysis. BMC Complement Altern Med.

[2] González RG, Furie KL, Goldmacher GV, Smith WS, Kamalian S, Payabvash S (2013). Good outcome rate of 35 % in IV-TPA-treated patients with computed tomography angiography confirmed severe anterior circulation occlusive stroke. Stroke..

[3] Chen J (2015). Progress of clinical research of acute cerebral infarction. J Clin Med Literat.

[4] Li HH, Hu ZL, Chen T, Dai ZW (2014). Dynamic changes of serum brain natriuretic peptide of patients with acute cerebral infarction and its clinical significance. Chongqing Med J..

[5] Shen JC (2011). Clinical significance of detection of levels of serum D-dimer and high-sensitivity C-reactive protein of patients with acute cerebral infarction. Lab Med..

[6] Ayhan H, Kasapkara HA, Durmaz T, Keles T, Sari C, Erdogan KE (2015). Evaluation of CA125 and NT-pro BNP values in patients undergoing transcatheter aortic valve implantation. J Geriatr Cardiol.

[7] (1996). Chinese Academy of Neurology. The essentials of diagnosis of cerebrovascular diseases. Chin J Neurol.

[8] Muchada M, Rubiera M, Rodriguez-Luna D, Pagola J, Flores A, Kallas J (2014). Baseline National Institutes of Health stroke scale-adjusted time window for intravenous tissue-type plasminogen activator in acute ischemic stroke. Stroke.

[9] Naoi H, Hashimoto H, Kajimoto E (2013). Cerebral infarctions as manifestation of ovarian clear cell carcinoma:report of two cases and review of the literature. Int Cancer Confer J. Springer Japan.

[10] Song LY, Zhou Y, Zhai WQ (2014). Discussion on detection of serum ORP, FG and LP(a) of elders with acute cerebral infarction and its clinical significance. J Hainan Med Univ.

[11] Amin AS, Peters RH, Verstraaten M, Wilde AA, Buijs EM (2015). Baseline NT-Pro BNP level predicts success of cardioversion of atrial fibrillation with flecainide. Neth Heart J.

[12] Kim SH, Lee JY, Park SH, Jang HC, Lim EJ, Chang SJ (2013). Plasma B-type natriuretic peptide level in patients with acute cerebral infarction according to infarction subtype and infarction volume. Int J Med Sri.

[13] Perlini S, Salinaro F, Perrone T (2015). NT-pro BNP and the risk of incident hypertension:is change over time a better predictor than baseline value?. J Hypertens.

[14] Chen SH, Li CP, Yu J, Gong JQ (2015). Study the effect of argatroban injection on serum copeptin, NT-pro BNP levels and clinical efficacy in patients with acute cerebral infarction. Chin J Biochem Pharm.

[15] Piao Y, Xu SJ, Wang Z, Wu LH, Liu N, Chen J (2014). Correlation between levels of NT-pro BNP and two D-dimer and injury degrees of cerebral infarction. J Apopl Nerv Dis.

[16] Ma T, Liu P, Guo X, Ding HR, Zhu YR, Liu X (2015). Clinical values of detection of plasma fibrinogen, D-dimer and NT-pro BNP of patients with cerebral infarction. World Clin Med.

[17] Xu SF, Xu YH (2013). Detection of homocysteine, D-dimer and fibrinogen of patients with acute cerebral infarction. J Clin Transfus Lab Med.

[18] Jiang K (2015). Analysis on the detection results of homocysteine and D-dimer of 76 patients with acute cerebral infarction. Med Inform.

[19] Ito H, Tanaka Y, Sase T, Uchida M, Yoshida Y, Sakakibara Y (2014). Cerebral hyperperfusion syndrome following the excision of a mycotic aneurysm with superficial temporal artery-to-middle cerebral artery bypass:case report. Neurol Med Chir (Tokyo).

[20] Matsumoto H, Kohno K (2011). Posttraumatic cerebral infarction due to progressive occlusion of the internal carotid artery after minor head injury in childhood:a case report. Childs Nerv Syst.

